# Re-Thinking Felid–Human Entanglements through the Lenses of Compassionate Conservation and Multispecies Studies

**DOI:** 10.3390/ani12212996

**Published:** 2022-10-31

**Authors:** Margarita Steinhardt, Susanne Pratt, Daniel Ramp

**Affiliations:** 1 TD School, University of Technology Sydney, Ultimo, NSW 2007, Australia; 2 Centre for Compassionate Conservation, TD School, University of Technology Sydney, Ultimo, NSW 2007, Australia

**Keywords:** compassionate conservation, multispecies studies, cohabitation, coexistence, felidae

## Abstract

**Simple Summary:**

Felids have long and complex historical associations with humans, ranging from fear and persecution to worship and care. With many felid species in widespread decline, re-thinking the messy entanglements of feline predators and human societies is a necessary step for fostering coexistence as current conservation frameworks that rely on the separation of people from nature are failing felids. Here, we explore two distinct but related interdisciplinary fields that, when put into dialogue with one another, offer novel perspectives and insights on felid–human relationships and conservation initiatives more broadly. We identified numerous similarities and emergent properties within compassionate conservation and multispecies studies, despite these fields arising from the sciences and social sciences and humanities respectively. Combined, reorientation of conservation values and practices to be morally inclusive of individual animals and their subjective experiences has the potential to support cohabitation and tolerance for felids, promoting multispecies flourishing.

**Abstract:**

With many felid species in widespread decline, re-thinking the messy felid–human entanglements is a necessary step for fostering coexistence as current conservation frameworks centered on human exceptionalism and widespread violence toward wild animals are conspicuously failing felids. This paper argues for fostering a critical awareness of how we understand our relationships with nonhuman animals, particularly in the context of conservation. We bring two distinct but related interdisciplinary fields into a dialogue to critically question the values and conceptual assumptions that frame the practices of felid conservation today. Compassionate conservation and multispecies studies share many synergies and conceptual overlaps despite emerging from different academic domains. We identified four key areas for further exploration: (1) A shift in emphasis from practices of killing to the underlying assumptions that make forms of killing permissible and ethically unproblematic. (2) Re-engagement with individuals, not just species, in conservation settings. (3) Unsettling human exceptionalism through an emphasis on the agency of animals and an ethic involving compassion. (4) Acknowledging the ways in which humans co-become with other animals and cultivating relationships of multispecies cohabitation and flourishing.

## 1. Introduction

Felid–human relationships have a long, complex, and dynamic history. Over the millennia, humans have feared felids as apex predators, worshipped them as gods and symbols of power, adored them as pets, exterminated them as pests, and protected them as embodiments of normative constructs of “pure” and “untamed” nature [1,2]. However, efforts to protect felids and other species are often grounded in problematic worldviews, particularly those that systematically separate humans from and elevate them above all other beings [3,4]. The belief in the exceptional moral status of “the Human” and associated exclusionary frameworks continues to dominate Western thinking and have profound effects on the lives and deaths of other animals. In the case of felids, their habitats have been transformed into human-dominated landscapes, their social structures disrupted, and their prey depleted and replaced with domestic animals. As felids strive to persist within these altered environments, they are routinely shot, poisoned, and left to die in snares in response to real or perceived threat their presence poses to human safety and livelihoods [5,6]. These killings often occur under the guise of protection, through practices of trophy hunting, predator control, or the preservation of genetic “purity” of a species [6,7,8,9,10]. With the global decline of felids, current approaches and frameworks centred on human exceptionalism and widespread violence toward wild animals are conspicuously failing felids. In response, there have been mounting calls across diverse academic fields for new paradigms of human-wildlife relationships, reshaping our responsibility towards other species [2,11,12,13,14,15]. 

Two key fields, emerging from different disciplines, that are directly engaging with re-imagining conservation paradigms are “multispecies studies” and “compassionate conservation”. However, efforts to advance these new paradigms are constrained by their basis in distinct academic fields, with limited cross-pollination across domains despite shared goals and conceptual overlaps [16]. By aligning disciplinary advances to enhance connectivity and synergies, novel and innovative approaches to coexisting with felids may arise and help conserve this faunal group from further decline.

Within the social sciences and humanities, the interdisciplinary field of multispecies studies explores ethical, political, and epistemological entanglements of humans and other beings, contending that animals’ entanglements with humans must be theoretically integrated into any accounts of existence [15,17]. The term “entanglements” highlights the messiness, spatiality, and materiality of people’s relationships with animals, and the power dynamics that constitute human-wildlife relationships in shared spaces [5]. Within science, the interdisciplinary field of compassionate conservation has been advocating for transforming conservation’s relationship with nature by expanding the discipline’s moral scope and considering animals as thinking, feeling, and agentive beings worthy of moral concern [18,19,20]. 

This article brings compassionate conservation and multispecies studies into a conversation to critically question the values and conceptual assumptions that frame the practices of felid conservation today and explore how these values can be transformed from the entrenched ontological dualisms towards a relational ontology attuned to the possibility of response-able felid–human cohabitation where response-ability is understood as “collective knowing and doing, an ecology of practices” [21]. The purpose of this cross-disciplinary dialogue is to leverage the conceptual similarities between these two fields and explore how they can help us interrogate the foundations of felid–human relationships, which may, in turn, lead to the emergence of novel and innovative solutions to the challenges of felid conservation.

The article proceeds as follows: The following section outlines different contexts of positive felid–human cohabitation and conservation, and positions this in relation to multispecies studies and compassionate conservation. The next section further situates this research by providing a brief outline of how conservation values have shifted since the inception of conservation paradigms to highlight the evolving tensions in the way people’s relations with nature and wild animals have been conceptualised. Next, compassionate conservation and multispecies studies are brought into dialogue to build a foundation of shared values driving a shift towards a relational ontology in conservation and felid–human relations. The conclusion highlights the potential for deeper engagement between these two bodies of scholarship and suggests directions for future research.

Before proceeding, it must be noted that this article is focused on exploring the specific lineages of conservation paradigms, and therefore it engages primarily with Western ontological and epistemological traditions and assumptions. However, while conservation paradigms emerged in the West, they have been exported around the world through the processes of globalisation and international conservation policymaking, shaping the possibilities for people’s relationships with wild animals worldwide [22].

## 2. Setting the Scene: Positive Felid–Human Cohabitation and Conservation

How animals are conceptualised matters not only because societal beliefs determine how humans treat other species but also because they shape conservation paradigms that, in turn, structure future possibilities for human–wildlife interactions [23]. Despite the growing body of scholarship in the social sciences and humanities seeking to replace dualist ontologies with relational perspectives and overcome anthropocentrism by recognising animal agency [17], traditional conservation has been slow to engage with novel conceptualisations of human-wildlife relationships [14]. Many contemporary conservation practices and policies are still firmly entrenched in the ontology of separation and human exceptionalism [2,14].

Across the sciences, social sciences, and humanities, particularly within multispecies studies and compassionate conservation, the concept of cohabitation is being proposed as an alternative to the dichotomous and anthropocentric frameworks that conceptualise nature and culture as radically separate realms [24,25,26]. The entanglements of human and animal lived experiences are emphasised in this emerging concept. The central premise of cohabitation is that people and wild animals have the capacity to change their behaviour and adjust to each other’s presence in the landscape as they learn to live together [24]. In contrast to the more-widely accepted framing of coexistence that is largely focused on managing and minimising negative impacts of humans on wildlife and vice versa [27], the concept of cohabitation speaks to transforming the foundations of human-wildlife relationships and recognising inseparable and interdependent entanglements of humans and all other beings and their environment. From the compassionate conservation perspective, peaceful felid–human cohabitation can be enacted when felids are valued and accepted by human communities as morally relevant inhabitants of the shared landscapes. In other words, positive felid-human cohabitation is predicated on moral inclusion which is situated in specific contexts [28].

Some of the diverse contexts in which examples of positive felid–human cohabitation can be found include urban environments, agricultural, ecotourism, citizen science and traditional ecological knowledge (see Table 1). By “positive” we mean mutualistic relationships where humans consider felids as “part of an extended family, and as deserving of caring and compassion” [29]. Situated in unique local contexts and histories, these relationships are fostered by recognition of felids’ agency in shaping local environments and behavioural opportunities for their human neighbours [30]. Humans respond by adapting to particular felid ecologies and behaviours and create new possibilities for felids to respond. This process of deeply situated dynamic co-becoming is what produces intimate felid–human entanglements.

**Table 1:** Positive felid–human entanglements in different contexts.

Context	Felid–Human Entanglement
Urban	Unlike in most American cities, where a wandering mountain lion (*Puma concolor*) would be shot on sight, in Los Angeles, mountain lions peacefully cohabit with humans on the fringes on the metropolis (Figure 1). Most Lost Angeles mountain lions occur in Santa Monica Mountains and avoid developed areas [31]. However, one male, known as P-22, settled in urban Griffith Park, a large urban park surrounded by residential neighbourhoods, a decade ago and has become a frequent feature on security cameras in the neighbourhood backyards. Instead of reacting with fear, local residents want to be good neighbours to the mountain lions [32]. P-22 became the symbol of human–wildlife cohabitation in urban landscapes and an inspiration for construction of the Wallis Annenberg Wildlife Crossing in Los Angeles – the world’s largest wildlife crossing to connect two areas of mountain lion habitat in the Santa Monica Mountain Range (Figure 1).
Agricultural	In the Brazilian Pantanal, Fazenda San Francisco, a 15,000-hectare working farm, successfully combines cattle ranching and wildlife tourism. Almost half of the farm’s area is set aside as wildlife habitat that supports populations of jaguars (*Panthera onca*) and ocelots (*Leopardus pardalis*) among other species, while the other half is split between cattle pasture and rice fields. Human-livestock-jaguar cohabitation is fostered by collaborative multispecies approach that includes the use of controlled breeding season for the cattle and establishment of mixed flocks of cattle and water buffalo in the maternity paddocks. Unlike the cattle, water buffalo are not intimidated by the jaguars and protect their young by forming a defensive circle around them. As a result, virtually no cattle are lost to predation at the farm [33] and the presence of jaguars and ocelots in particular draws a steady stream of eco-tourists to the farm (Figure 2).
Citizen science & conservation	In Amboseli-Tsavo Ecosystem in southern Kenya Lion Guardians program is transforming the relationships between Maasai ranchers and lions (*Panthera leo*). The program employs traditional warriors to transfer their skills from killing lions to collecting and reporting data to the scientists, alerting herders to lion presence to prevent attacks on livestock, locating livestock lost in the bush and improving livestock husbandry [34]. The guardians give traditional Maasai names to the lions they identify and share stories about ‘their’ lions with the broader community. This practice of personalising lions through storytelling transformed the lions from anonymous enemies to recognisable individuals even for the community members not directly involved in their conservation. As nonhuman persons [35] lions became morally relevant neighbours which led not only to a dramatic decrease in lion killings by the Maasai but also to a two-fold increase in lion population in the region.
Ecotourism	Russian Far East where a single Siberian tiger’s (*Panthera tigris altaica*) territory can exceed 1385 km [36], has one of the lowest human population densities in the world. To reverse depopulation in the region, Russian government offers inexpensive long-term land leases to entrepreneurial individuals who can profit from their plots by selling hardwood trees, hunting, trapping, fishing or prospecting for gold. However, since the arrival of the international eco-tourism company, Royle Safaris and establishment of small-scale ecotourism in the region, more and more lease holders are setting up their land as tiger reserves, transforming their relationship to the forest. Instead of extracting forest products from their plots, many lease holders buy grain to attract tiger’s prey species back to the area and, in collaboration with an NGO established by Royle Safari, plant trees to reforest previously logged areas [37]. Human-tiger cohabitation is fostered by keeping ecotourism activity at small, sustainable scales to minimise disturbance to the tigers and foster future encounter opportunities (Figure 3).
Traditional Ecological Knowledge	In the Romanian Carpathian Mountains large carnivores, including the Eurasian lynx (*Lynx lynx*) —Europe’s largest felid, share space with humans and livestock. The relatively peaceful cohabitation in the region is fostered by the uninterrupted use of traditional livestock guarding dogs by farmers and shepherds, since the beginning of pastoral activity in the Carpathians. Drawing on centuries of traditional ecological knowledge and relying on the dogs’ keen senses local shepherds avoid negative encounters with carnivores and keep their flocks safe from predation. The presence of carnivores is seen as a fact of life in the region. The multi-generational relationships of response-able cohabitation between Carpathian shepherds and carnivores may be the reason why Carpathian Mountains still support one of the highest densities of large carnivores in Europe [38].

The place-specific variability in felid–human cohabitation opens up a series of questions such as, why are some felid–human communities able to flourish while others are fraught with conflict? When and how do lived experiences of felids and humans intersect? What cultural and biological traits foster response-able cohabitation? Furthermore, what is the role of conservation in shaping these relationships?

These questions are at the heart of compassionate conservation and multispecies studies scholarship. While these two fields emerged from distinct conceptual and epistemological traditions, they share deep affinities in how they engage with the more-than-human world. Compassionate conservationists and multispecies studies scholars share an interest in how humans and wild animals relate to one another, emphasising the lived experiences of individual animals rather than privileging the abstract categories of species and populations. Both scholarships problematise the notion of “management” of wild animals by considering animals not as passive objects being acted upon by humans, but as agentive beings actively co-shaping the spaces in which they dwell and, to some extent, co-shaping conservation practices [13,39,40,41]. The striking conceptual parallels between compassionate conservation and multispecies studies and their shared foundations in relational worldviews have been exemplified in recent scholarship that draws on the elements of both fields [35,42,43,44]. We build on this earlier work by explicitly drawing out conceptual connections between multispecies studies and compassionate conservation as this has been underexplored [with the notable exception of [40,41]. From this transdisciplinary perspective, the converging lines of enquiry in compassionate conservation and multispecies studies highlight the need for establishing the common ground between these two bodies of scholarship [45]. Before delving into deeper commonalities and differences between these two fields we next provide a brief environmental history of felid–human relations, including a contemporary focus on conservation, to offer further context for the comparison.

## 3. An Environmental History Perspective of Felid–Human Relations and Shifting Conservation Paradigms

Humans and felids have co-shaped their shared worlds for as long as there have been humans. Ancestral felids were part of the multispecies landscape in which *Homo sapiens* evolved. While the prehistory of the ecological relationships between early humans and various species of felids is obscured in the fragmented and incomplete fossil record, there is little doubt that as apex predators, felids featured prominently in the lives of our ancestors. Over the millennia, felid-human entanglements transcended the physical realm of mortal struggle and found their way into art, literature, mythology, and religion embodied in mystical figures such as the Egyptian lion-headed goddess Sekhmet [46], the half-man-half-lion Indian god Narasimha [47], were-jaguars of Meso America [48], and the felinised creatures such as the sphinx, griffin, and chimera [49]. What these cultural representations have in common, beyond expressions of fear, respect, and admiration of felids, are the relationships of kinship between human societies and powerful feline predators, relationships in which notions of personhood were shared by humans and felids [50]. Throughout history, humans have tolerated the presence of dangerous predators in the landscape, finding roles for them in our cultural and spiritual realms [51].

However, as human societies transitioned to agricultural lifestyles, they adopted more anthropocentric perspectives on wildlife [52]. Some animals were incorporated directly into these agricultural lifestyles as pets or livestock, while others, including the majority of felid species, were excluded as threats to human safety and livelihoods [52]. The turning point in human-wildlife relationships came around the time of the Renaissance, with the emergence of “anthropocentrist humanism” in Europe–a belief system based on “conceptualizing human being over and against animal being, and privileging human consciousness and freedom as the center, agent, and pinnacle of history and existence” [53]. Societies that adopted renaissance values no longer saw themselves as part of ecological relationships, and the danger that the large predators posed to human livelihoods and expressions of evolutionary superiority and progress became unacceptable. Consequently, centuries of ensuing development associated with European colonisation of the Americas, Africa, and Asia were marked by the systematic eradication of predators, and the majority of other nonhuman forms of life, from the landscape [54,55].

However, as multispecies scholars remind us, “life and death do not take place in isolation from others, they are thoroughly relational affairs for fleshy, mortal creatures” [56]. Felids were far from a static background against which human dramas unfolded. Like other species of carnivores [40,57], felids shaped their own lives and the lives of humans by adapting to the new dynamics of their changing environment and learning to use the new features to their advantage. The best example of complex ecocultural entanglements between humans and felids is that of the wildcat (*Felis silvestris*). This small hunter of rodents exploited a unique niche provided by the early agricultural settlements and over time established deep social ties with human societies and became the most popular companion species in human societies worldwide [58]. Other species of felids learned to live in human-dominated landscapes without coming into direct contact with them. In some places, leopards (*Panthera pardus*) and pumas (*Panthera concolor*), for example, have learned to adjust their movements and activity patterns to avoid encountering humans living in the same landscapes [59,60]. In other places, some species of felids turned to hunting humans as prey. Some of the most notorious examples of man-eating behaviour have been documented for tigers in the Sundarbans [61] and Kumaon [62] in India, and lions in Tsavo in Africa [63]. The localised nature of these behavioural traditions in felids highlights the importance of local contexts in felid–human relationships [63] and implies that some of these predators developed cultures of preying on humans [64]. Animal culture is understood here as “information or behaviour shared within a community which is acquired from conspecifics through some form of social learning” [65]. Beyond these exceptional occurrences, felids and humans are not enemies, and for the most part, most cats avoid contact with humans and retreat at the first sign of human presence. However, the expansion of pastoralism around the world and the associated depletion of natural prey and conversion of wildlife habitats to human-dominated landscapes coupled with humanist worldviews of the colonial settlers introduced a new type of relationship between humans and felids, one of “felid-livestock-human” entanglements [66], which remain one of the biggest challenges for response-able felid–human cohabitation.

Within contemporary narratives, encounters with wild cats are predominately shaped by conservation context, where even the notion of a “species” is reflective of protective responsibilities and actions. Consequently, conservation policies shape certain kinds of felid-human relationships and therefore structure the possibilities for human-wildlife interactions. Yet, despite conservation’s noble goal of protecting the earth’s biodiversity, the discipline has been criticised for being rooted in the dualistic ontology that has arguably led to the global environmental crisis [2,14]. While the framing of conservation has shifted over the decades [67], the underlying ontological distinctions between nature and culture, human and nonhuman, subject and object, remain entrenched in how the relationships between people and nature are viewed. Furthermore, as Nustad points out, “if the ontological distinction between nature and humanity evolved as a reaction to the destruction of the environments, can that same ontology form part of the solution?” [1]. 

As with all scientific knowledge, the science of conservation is fundamentally shaped by social relations and practices [68]. Perhaps, one of the fundamental reasons for our inaction is our failure to fully comprehend the meaning of the global environmental crisis [69]. The following sections of this article introduce two growing bodies of scholarship that have emerged from different disciplines—multispecies studies and compassionate conservation—which are informing contemporary debates around conservation. It brings these two bodies of scholarship into conversation with each other to explore points of connection and contrast in the way they engage with the shift towards a relational ontology that informs emerging practices surrounding conservation and felid–human relations.

## 4. Theoretical Lenses of Multispecies Studies and Compassionate Conservation

### 4.1. Multispecies Studies

Multispecies studies are an umbrella term proposed by van Dooren, Kirksey & Münster [15] to bring together diverse disciplinary and interdisciplinary approaches that have emerged in recent years in the social sciences and humanities, including multispecies ethnography, anthropology of life, anthropology beyond humanity, more-than-human geographies, and extinction studies. Despite their differences, these fields are united by an interest in exploring ethical, political, and epistemological entanglements of humans and other beings, primarily by employing a methodological approach that Anna Tsing defined as “passionate immersion in the lives of nonhumans being studied” [70].

A core feature across many multispecies studies scholars’ work is a rejection of any absolute boundaries between ontology and epistemology in favour of a relational worldview that Karen Barad [71] termed “agential realism”. From this perspective, the world emerges as materially real through a process of continuing “intra-actions”, a process that recognises that individual entities, things, and concepts emerge through their entangled intra-relating [71]. This relational worldview underpins the framing of felid–human cohabitation by conceptualising felid–human communities as continuous negotiations of interspecies co-becoming.

Although van Dooren, Kirksey & Münster [15] do not directly engage with multispecies justice in their overview of multispecies studies, it is important to briefly highlight the relationship of these terms, as they are sometimes incorrectly used interchangeably. In this article, multispecies justice is conceptualised as a focus area, informed strongly by justice theory and practice, within the broader field of multispecies studies. As Celermajer et al. [72] articulate, multispecies justice “seeks to understand the types of relationships humans ought to cultivate with more-than-human beings so as to produce *just* outcomes.” So, while both multispecies studies and multispecies justice strive for more relational ontologies, the broader field of multispecies studies is not always centred on producing just outcomes, such as legislative change. We use, and focus on, the broader term of multispecies studies here, as there is limited scholarship comparing compassionate conservation and multispecies studies [however see 40,41]. There is scope for future work to then narrow in, with a justice lens, to compare multispecies justice to compassionate conservation.

### 4.2. Compassionate Conservation

The concept of compassionate conservation emerged from growing tensions within the conservation community around conservation’s goal of achieving the greater good for the Earth’s biodiversity and its over-reliance on lethal management strategies to achieve that goal, coming at the cost of the welfare and wellbeing of animals. Motivated by the virtue of compassion, and underpinned by mounting scientific evidence of animal sentience and sapience, compassionate conservation arose as a challenge to the narratives of traditional conservation that separate living beings into the hierarchies of those who belong within the moral circle of conservation and those who do not; those that deserve protection and those whose lives are sacrificed for this protection [13,19].

While early work in the field of compassionate conservation was motivated by the pragmatic aim of inspiring change in conservation practice towards prioritising nonlethal and noninvasive strategies in conflict resolution [73,74], recent work has advanced compassionate conservation beyond this pragmatic root to engage with broader questions of what counts as nature in conservation and how we understand our relationship with and responsibility towards all other living beings on Earth [18,35]. In this way, compassionate conservation has instigated paradigm change within conservation by exploring the context for why and how individual lives are subjugated by concern for species and ecosystems, and in doing so, advocating for a relational understanding of compassion as an experience of recognition and of ‘suffering with’ others [35,43]. Drawing on the concept of ecological interconnectedness, compassionate conservationists view all beings as ‘beings-in-relation’ and compassion as an affective relationship of our interdependence [43]. From this perspective, compassion recognises nonhuman others as fellow persons who are "intrinsically and uniquely valuable” [35], creating entangled relationships of co-becoming [35,43].

Compassionate conservation has certain foundational values and beliefs, but it is not prescriptive in its nature and allows ‘a degree of pluralism in values and scientific judgement regarding animals and conservation practice’ [20]. It offers a set of guiding principles that are articulated as the four foundational tenets: (1) first, do no harm—pause from harmful interventions; (2) individuals matter—lives of individual animals have intrinsic value; (3) inclusivity—all individuals and collectives have intrinsic value; and (4) peaceful coexistence—in instances of conflict, first consider modifying human practices and fostering a culture of coexistence [13].

## 5. Multispecies Studies and Compassionate Conservation in Dialogue

### 5.1. From Killing to Making Killable

Millions of animals are killed each year worldwide in the name of conservation [19]. Compassionate conservation and multispecies studies are deeply concerned with this harm and violence in conservation practice, although their approaches differ. Compassionate conservation’s first tenet, ‘first, do no harm’ recognises that human interventions in ecosystems can cause more harm than good to animals and counsels the conservation community to carefully weigh any decisions to intervene in situations of conservation concern [13]. This tenet calls for a pause from intervention to enable the values that underpin the other three tenets of compassionate conservation to be carefully considered. It would, therefore, be problematic to engage with this tenet as a stand-alone concept, out of context of the other tenets, but it may be useful to do so in order to draw out productive tensions between the approaches of compassionate conservation and multispecies studies in relation to the notions of harm and ‘killability’ [75].

Adapted from medical bioethics, this tenet was formulated with the pragmatic aim of bringing together stakeholders with diverse views and perspectives and providing a common language for considering ethical ramifications of conservation’s reliance on lethal strategies in addressing conservation conflicts [74]. At face value, the articulation of this tenet appears to maintain the anthropocentric view of humans as independent change agents or managers of ecosystems that stand apart from the rest of nature.

Multispecies scholars have long taken seriously the notion of killability embedded in our relationships with nonhuman others. Haraway [75], for example, cautioned against separating beings into those that may be killed and those that may not and against pretending to live outside of killing. She argued that: “try as we might to distance ourselves, there is no way of living that is not also a way of someone, not something, else dying deferentially …. It is not killing that gets us into exterminism, but making beings killable” [75]. In other words, the only way to avoid killing is to consider some lives invisible [76], non-existent in the first place. Therefore, Haraway suggested abandoning the anthropocentric commandment “Thou shalt not kill” in favour of a commandment more attuned to the practices of nurturing and killing as an inescapable part of multispecies entanglements—“Thou shalt not make killable” [75]. Similarly, Plumwood [4] problematised the categories of killability in her gripping account of surviving a crocodile attack in Kakadu National Park, Australia. Plumwood surmised that seeing oneself as part of the food chain, as human prey, collapses the dualistic worldview in which humans see themselves as manipulating nature from the outside [4].

What the concept of killability allows us to do is to shift focus from the acts of violence against more-than-human others to the underpinning dualistic ontology that makes these acts permissible, changing the question from which beings are being killed to which beings are made killable [77]. In contrast to these accounts, compassionate conservation’s “first, do no harm” tenet, when considered as a stand-alone principle, can be interpreted as re-enforcing the human-nonhuman dichotomy. However, when we interpret this tenet as a pause that opens the space for the other three tenets to come in, compassionate conservation actively engages with categories of killability within the context of conservation science.

Animals are made killable in many ways in conservation, and it is in challenging these practices that work in compassionate conservation, and multispecies studies, align most closely. Compassionate conservation identifies and calls into question three ethical orientations that underpin the practices of making beings killable in the name of conservation: collectivism–species matter more than individuals; instrumentalism–individuals may be treated as means to an end; and nativism–populations established by humans are unnatural [13]. For example, a nativism orientation renders a large proportion of Earth’s biodiversity, including large terrestrial mammals, invisible (and therefore killable) in our accounts of nature because these animals occur outside of their native ranges and considered to be external to biodiversity [35]. Similarly, instrumentalism allows individual animals to be sacrificed for the perceived benefit of the species through such practices as trophy hunting [7]. As a counter view to human exceptionalism, compassionate conservation offers a more inclusive perspective by advocating for the inclusion of nonhuman animals in conservation’s moral community and recognising all sentient beings as persons [35,78].

However, a question remains in conservation practice whether some lives are more killable than others. Compassionate conservation advocates for creative alternative solutions to conservation conflicts [79], however, such solutions may not always be available. Haraway argued that we must learn to “kill well”, learn to live responsibly with the necessity of killing, and cultivate “capacity to respond in … multispecies contingency” [75]. Similarly, compassionate conservation recognises that sometimes many of the available courses of action involve a measure of wrongdoing and choosing any action leaves a “moral residue” that should be experienced as some form of grief [44]. Echoing Haraway’s explorations of shared suffering between scientists and their companion species of laboratory animals, compassionate conservation’s approach posits that feelings of grief are an appropriate response to the acts of harm and that conservationists owe “the honor of acknowledgement to the victims of their decisions” [44].

In traditional conservation, nature protection is framed as fundamentally about people making decisions on behalf of animals [41]. By shifting focus from the practices of killing to the underpinning assumptions that make these practices permissible, compassionate conservation opens the space within conservation science for asking questions that come more from the place of compassion and kinship and less from the position of control, domination, and decision making [41]. This work joins multispecies scholarship in challenging the narratives of traditional conservation that make the practices of killing appear “inevitable, unavoidable and ethically unproblematic” [80] and make the killing of undesirable others much easier than trying to figure out how to live with them [73,74]. The following section explores the values, beliefs, and assumptions embedded in traditional conservation discourse that sustain the practices of making beings killable.

### 5.2. From Species to Individuals: Who Lives, Who Dies, and Why?

The Canada lynx (*Lynx canadensis*) is a medium-sized cold-adapted felid that occurs across Alaska and Canada, as well as the northern areas of the contiguous United States where it is listed as a threatened species under the Endangered Species Act [81]. As part of a species recovery program, 218 Canada lynx were trapped in Alaska and Canada, transported to Colorado, placed in captivity, and then released in the San Juan Mountains of south-western Colorado between 1999 and 2006 [82]. By 2007, more than 100 lynxes had died either from the acute stress of capture and relocation, the stress of establishing territories in the new and unfamiliar environment, starvation, from being shot, or hit by vehicles. These animals perished in the name of establishing a self-sustaining population of Canada lynx in Colorado, following the extirpation of lynx from the state in the late 1970s. The reintroduction program was considered a success [82], and 101 lynx deaths were accepted as a sacrifice for the greater good of their species.

Such disregard for animal lives is permissible in the context of conservation practice because individual animals are excluded from the moral scope of conservation [13,18,35]. In conservation biology, the species is the “foundational ontological unit” for knowing and valuing life [83]. The ordering of species in vast lists, like the IUCN Red List of Threatened Species, and an increasing number of national endangered species lists, is the main tool for monitoring and protecting biodiversity [10,83]. In the context of species-based conservation, individual animals are regarded as no more than “instances of their type” [84], their moral significance subsumed to the category of the species, and their lives and deaths gain meaning only when they advance the wellbeing of the species [10].

In contrast, compassionate conservation recognises the intrinsic value of individual animals as well as species and ecosystems. Its tenet “individuals matter” advocates for expanding the moral circle of conservation to include all sentient beings that make up the Earth’s biodiversity [13,19]. Attuned to the notions of more-than-human subjectivity and agency, compassionate conservation urges the conservation community to recognise all sentient beings as persons [35]. Such recognition of subjectivity and individuality of animal beings redefines the possibilities of multispecies relationality. As Smuts [85] reflected after spending two years among the baboons in East Africa: “My awareness of the individuality of all beings, and of the capacity of at least some beings to respond to the individuality in me, transforms the world into a universe replete with opportunities to develop personal relationships of all kinds.” However, the call to incorporate the interests and welfare of individual animals in conservation discourse has been highly divisive. Critics of compassionate conservation expressed concern that if conservation was to embody care for individuals it would jeopardise the discipline’s goal of protecting the diversity and complexity of the Earth’s ecosystems [43]. In other words, caring for individuals is placed in opposition to caring for ecological collectives and biodiversity as a whole. Such dualistic thinking entrenched in traditional conservation narratives creates powerful hierarchies of valued and unvalued lives.

Multispecies scholars have also explored how conservation discourses marginalise the wellbeing of individuals and those who care for them [8,10]. For example, McCubbin and Van Patter [8] examined how the hierarchies of scale (ecosystem/individual), knowledge (reason/emotion), and gender (masculine/feminine) are embodied in the two contrasting figures - the othered Crazy Cat Lady and the privileged Trophy Hunter. Echoing recent work in compassionate conservation [43], they illustrate how mainstream conservation discourse positions care for individual animals as feminine and emotional while privileging species-based conservation as masculine and rational. Drawing on feminist environmental scholarship, McCabbin and Van Patter trace how the narrative of public outrage over trophy hunting in the wake of killing of Cecil the lion in Zimbabwe in 2015 was reshaped from concern for individual lions killed as trophies to a broad concern for lions in general. Silencing the emotional public outcry, conservation biologists argued that the issue of trophy hunting ought to be approached rationally, through science. In the context of species-based conservation, trophy hunting is presented in a positive light as a means of habitat protection and a source of funding for conservation of the species. From this perspective, the masculine figure of Trophy Hunter emerges as a rational conservation actor. In contrast, public concern for “feral” cats—cats that live “in the wild and can survive without human reliance or contact” [86]—is often aligned pejoratively with the feminine and a misguided expression of feminine emotional attachment, as encapsulated by the figure of the Crazy Cat Lady [8]. The following section takes a closer look at the paradoxical entanglements of humans and “feral” domestic cats to highlight some of the consequences of a species-focused approach to conservation. Following Fredriksen [10], inverted comas are used around “introduced”, “native”, and “feral” within this text to signal that these descriptors are more useful for denoting the human framings of which beings belong in certain landscapes and which do not, than for describing inherent qualities of these beings.

### 5.3. Wild, Feral, Human: Hierarchies of Value in Species Focused Conservation

Archaeological evidence suggests that felid–human relationships began during the emergence of agricultural settlements in the Fertile Crescent about 10,000 years ago [87]. Initially, wildcats (*Felis sylvestris)* were probably attracted to human settlements by the rodents that exploited the supplies of stored grains. The cats’ ability to catch mice and rats made them a useful companion for the early agriculturalists. By around 4000 years ago, wildcats were domesticated in Ancient Egypt, where they were considered one of the most sacred animals [87]. Over the following millennia, cats became one of the most popular companion species in human societies, with more than 370 million cats living alongside people across the world today [88].

However, when domestic cats transgress the boundaries of the domestic sphere and return to the “wild-living” lifestyle of their ancestors, albeit in novel environments, they are transformed from valued pets to “feral” pests [89], causing a “moral panic over cats” in conservation community [90]. Some of the most salient examples of “feral” cats’ entanglements with conservation come from Australia and Scotland, where cats are actively persecuted in the name of conservation. In Australia, domestic cats arrived with European settlers in 1788 and came to prominence a century later as “the enemy of the rabbit” [91]. In hopes that cat predation would control the spread of the rabbits, thousands of cats were released in Australia and given legal protection against being killed or captured [91].

Less than a century later, predation by cats emerged as a key threatening process to “native” biodiversity, and the cat was re-classified as an “invasive” species targeted by eradication programs. In 2015, the Australian government committed to killing 2 million “feral” cats by 2020 as part of the country’s first Threatened Species Strategy [92]. Despite an emotional public outcry that compared the government’s decision to animal genocide, an estimated 211,560 cats were killed during the first 12 months of the program [93].

While in the Australian context, the cat’s crime against conservation is predation on “native” species, in Scotland, the cat is persecuted for hybridising with its conspecific – the Scottish wildcat (*Felis silvestris grampia*). The Scottish wildcat is the last surviving member of the Felidae family in Britain, and with as few as 400 individuals left to roam the Scottish Highlands, it is considered one of the most endangered species in the UK [94]. After being persecuted by game wardens for centuries, the Scottish wildcat emerged from obscurity in the late 20th century as an “idealized wild embodiment” and conservation icon [95]. Furthermore, while the archaeological evidence suggests that Scottish wildcats have been potentially sympatric and hybridizing with domestic cats for 1200–1500 years [87], those charged with wildcat’s conservation consider the presence of “feral” cat in the Scottish Highlands to be a key threat to the survival of the genetically “pure” Scottish wildcat. As Fredriksen [10] notes, “In the context of species-based conservation, the difference between Scottish wildcats and ‘feral’ domestic cats–undoubtedly of little concern to the cats themselves, not to mention to the rodents and rabbits they prey on - gains salience” [10]. In species-based conservation discourse, the lives of wildcats become highly valued while their free-roaming feline cohabitants and hybrid offspring are rendered valueless threats to conservation of the species and, therefore, killable [10].

What emerges in both Australian and Scottish contexts is that species focused conservation not only elevates species over individuals but also creates hierarchies of valued and unvalued species and seeks to preserve the current assemblage of valued species on Earth [10]. Underpinned by ontological separation of humanity from the rest of nature and the framing of the environment “as a space to be thought about and managed by conservation objectives” [80], conservation discourse frames wildlife populations influenced by humans as “unnatural”, as if humans have the power to transform “natural” entities into “unnatural” ones [35]. As Ogden, Hall, and Tanita [96] argue, “If ecosystems are, as ecological theory posits, complex and dynamic assemblages of multiple species, including humans, then attempts to eradicate newcomers, often defined by a fairly arbitrary colonial timeline, speaks more to landscape nostalgia than science” [96]. From this perspective, the practices of killing “feral” cats in Scotland and Australia emerge as “aesthetic culls” aimed at producing ecologies desirable by humans [80]. The inclusivity implied by the term “biodiversity” has been lost as the science and practice of biodiversity conservation devolves into a distinctly exclusionary framework [10,28,80].

**Figure 4:**
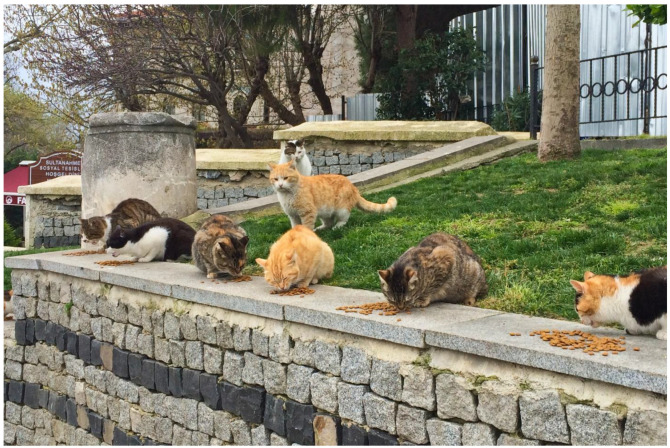
How we conceptualise animals matters. For example, in Istanbul, “feral” cats are seen as constituent inhabitants of the city, valued, and cared for by their human neighbours.

As a counter narrative to exclusion, compassionate conservation’s tenet of “inclusivity” embodies the intrinsic value of all living organisms regardless of their relationships with humans and call for “decentring humans from the stories of nonhuman persons” [35]. By interpreting compassion as an experience of suffering, or more broadly enduring, with others, compassionate conservation challenges the exclusionary practices that underpin traditional conservation and asks what happens if we reframe the question of “how can biodiversity be protected from feral cats with nonlethal tools” to “what is revealed when the feral cat is accepted as part of biodiversity” [35,97]. If conservation is to embody an ethic of compassion, then perhaps its values and principles could be best expressed by relational ontology, as ways of being in the world, grounded in curiosity and an interest in becoming with more-than-human others (Figure 4) rather than as a set of conservation objectives and desired outcomes [41,43].

Similarly, multispecies studies scholars problematise the hierarchies embedded in species-based conservation and conceptualise species as “vast intergenerational lineages, interwoven in rich patterns of co-becoming with others” [56]. From the perspective of multispecies relationality, “feral” cats emerge as “ontological amphibians” that move freely among worlds, continually entangling themselves in novel multispecies assemblages [98]. Recognition of others as agentive beings with their own interests and ways of being in the world draws us into a greater sense of accountability in our relationships with them. If we reject ontological dualisms that are at the core of human exceptionalism, then perhaps the goal of conservation may be expressed as learning to “value and care for what is here now, in a way that acknowledges that any given species or ecosystem, while being immensely valuable and precious in itself, is nonetheless a transitory and changing affair” [80].

### 5.4. Towards Response-able Multispecies Cohabitation 

The relational perspectives offered by compassionate conservation and multispecies studies are exemplified in the concept of multispecies cohabitation that both fields have engaged with in their unique ways [24,99]. The central premise of cohabitation is that people and wild animals have the capacity to change their behaviour and adjust to each other’s presence in the landscape as they learn to live together [24]. It builds on the more-widely accepted framing of coexistence and speaks to transforming the foundations of human-wildlife relationships and shifting the focus from mitigating human-wildlife conflict to re-thinking what is considered as conflict in conservation in the first place.

The term coexistence is widely used in conservation literature, however, in contrast to a relational understanding, it is typically framed as a set of conflict mitigation strategies aimed at protecting wildlife from human impacts or protecting human livelihoods from the negative impacts of wildlife [27]. This is a consistent pattern evident in the evolving framings of conservation [67], one that centres around the fundamental ontological distinction between nature and society. One of the knowledge-making practices that sustain these dichotomies in conservation discourse is the work of the “purification” of nature [2]. The idea of “pure” nature is grounded, on the one hand, in the romantic longing for landscapes that have not been dramatically altered by industrialisation, for a nature pure and pristine, free of human agency [1]. On the other hand, the work of “purification” reflects the practices embedded in the creation of scientific knowledge, the way phenomena are divided into “pure” categories of nature and society, human and nonhuman [2]. In other words, “pure nature” is a normative construct rather than an intrinsic property of any landscapes [2,100].

This construct is particularly relevant to conservation of large felids as it conceptually associates dangerous carnivores with “purified” environments of protected areas, effectively excluding the possibility of humans and large felids coexisting together [2]. Many large felids live outside of protected areas, but since they are conceptually framed as being out of place in multiuse landscapes, their presence is often perceived as conflict to human interests. In contrast to the notion of “pure” nature, multispecies scholars and compassionate conservationists ask us to imagine an “impure nature”, a type of hybrid environment shaped by the long histories of humans, animals, and plants all acting together to co-shape shared worlds [1].

In recent years, a new framing of human-wildlife interactions has emerged in conservation literature that adopts a more relational perspective, defining coexistence as “a sustainable though dynamic state, where humans and wildlife coadapt to sharing landscapes and human interactions with wildlife are effectively governed to ensure wildlife populations persist in socially legitimate ways that ensure tolerable risk levels” [101]. In this context, adaptation denotes the capacity of humans and other animals to change their behaviour and adjust to each other’s presence in the landscape. There are, indeed, numerous examples of felids adapting to human-modified landscapes. In the Indian state of Maharashtra, leopards living in close proximity to humans in highly modified agricultural landscapes learned to avoid direct contact with people by moving away from settlements during the day when human activity is highest, then returning to the villages at night when human activity is low [59]. Similarly, tigers share landscapes with humans outside Chitwan National Park in Nepal by adjusting their activity patterns to be less active during the day when human activity is highest [102]. In California, mountain lions move through human-modified landscapes by using riparian habitats and speed up when they have to traverse human-dominated areas to reduce the time spent in these areas [60].

However, while the framing of coexistence as a dynamic process of mutual adaptation is attuned to animal agency in that it recognises animals’ capacity to learn and adapt, it does not engage with questions of how wild animals are conceptualised - a vital step in fostering relationships of mutual adaptation. This is where the concept of cohabitation extends the framing of coexistence to conceptualise animals as fellow inhabitants who actively co-shape or co-produce the spaces where humans and animals can dwell [24]. As examples in this article demonstrate (Table 1), positive relationships of felid–human cohabitation are possible when felids are valued and accepted by human communities. In relationships of cohabitation, felids are transformed from unwanted trespassers to respected neighbours with their particular ecologies and behaviours. Mutual adaptation means that it is not only felids who adjust, but that humans change their practices too. Just as cultural narratives about nature shape societal beliefs about which beings belong where, the stories we tell about wild animals also shape what is considered as conflict in human-wildlife relations.

The embodied and embedded specificity of felid–human entanglements highlights the importance of animal cultures and place-specific histories of felids interactions with their human neighbours [65]. The ability to acquire knowledge from conspecifics through social learning may play an important role in felids’ ability to adapt to their changing environments. While felids are typically considered solitary animals, with the exception of lions and “feral” cats, they live within the social context of partially overlapping home ranges and use a wide range of olfactory signals to communicate with their neighbours by way of leaving scent marks throughout their home ranges. With its attentiveness to the lived experiences of wild animals, compassionate conservation extends animal cultures scholarship by incorporating insights from the field of animal cognition into ecological research. For example, Wooster et al. [103] invite us to re-imagine predator-prey interactions as taking place in a “landscape of knowledge” where predators and prey coexist and shape their shared worlds based on their knowledge of each other. Similarly, multispecies studies scholars conceptualise the practices of acquiring interspecies knowledge between humans and their more-than-human neighbours as a process of multisensory reading and writing where each species leave their own signs on the landscape and interpret the signs left by others [24].

To advance this line of inquiry and gain a deeper understanding of how humans and multiple other species co-construct their shared worlds, in-depth explorations of particular and deeply situated relationships between human communities and wild felids are needed. Multispecies studies, and especially the methodological approach of multispecies ethnography, are well placed to reframe studies of felid–human coexistence as studies of felid–human communities [104]. Highlighting rich relational narratives focused on place-specific values, histories, and adaptations that sustain relationships between people and potentially dangerous carnivores, multispecies ethnographers have explored tiger-human communities in India [105], hyena-human communities in Ethiopia [57], dingo-human communities in Australia [106], and crocodile-human communities in Florida Everglades [107]. While deeply attuned to the global political and economic forces that shape local contexts, this work also embraces the nuance and the multiplicity of worldviews, values, and ways of being in the world that are grounded in the local place-based histories and relationships within single communities.

## 6. Multispecies Studies and Compassionate Conservation in Context and New Directions

Making sense of felid–human entanglements is inextricably linked with the dominant discourse of conservation and the ways in which this discourse shapes human relationships with the more-than-human world. In recent years, the ontological, epistemological, and ethical foundations of conservation have been brought into question not only in the social sciences and humanities [1,80] but within the natural sciences as well [2]. As part of this trajectory, the fields of compassionate conservation and multispecies studies seek to rethink our relationship with nature. They replace normative conceptual frameworks based on the radical separation of nature and society and the associated belief in human exceptionalism with relational perspectives attuned to more-than-human agency and subjectivity.

These two bodies of scholarship are evolving simultaneously within their respective disciplines, however, the relationships between these two emerging fields and their parent disciplines have been markedly different. One key contrast is that while multispecies studies is becoming increasingly mainstream within the social sciences and humanities, pushing the scholarship in new directions [22,72], compassionate conservation’s call to incorporate the interests and welfare of individual animals in conservation science and practice has led to a heated debate in conservation literature [for review see 20]. If conservation is to transform its relationship to nature and animals, the impetus may have to “come from within the halls of science” [78], but this effort would be strengthened by collaboration with disciplines that have a history of ontological analysis and questioning. Compassionate conservation opens the space for such collaboration to take place.

This article has laid some of the foundations for how multispecies studies and compassionate conservation can build practices and knowledge across their respective disciplines within humanities and science. Further sharing of methodologies and deeper conceptual integration of multispecies relationality and compassion as a way of relating to other-than-human beings, across these two fields, would strengthen the interdisciplinary efforts to dismantle human exceptionalism and flatten the hierarchies and dichotomies embedded in traditional conservation discourse.

One of the most promising areas of collaboration between compassionate conservation and multispecies studies could be the development of shared methodologies for understanding the subjective experiences of animals. Both multispecies scholars and conservation scientists have called for a greater integration of scientific methods with multispecies ethnographic methodologies [40,64,104,108,109,110], proposing, for example, the use of monitoring technologies for tracking animal cultures, and the use of ethnographic case study approaches in studies of carnivore-human coexistence [104]. What multispecies scholarship brings to the study of animals’ subjective worlds is the language and the theoretical framework, while compassionate conservation contributes the field skills, tools, and technologies, as well as in-depth knowledge of animal biology and behaviour.

It must be noted that the recent work that mobilises relational ontologies in conservation practice is indebted to Indigenous knowledge, scholarship, and worldviews [111,112]. While it is beyond the scope of this article to explore this important body of scholarship and its integration with multispecies studies and compassionate conservation, it remains an important direction for future research for both scholarships.

## 7. Conclusions

This article brought multispecies studies and compassionate conservation into dialogue to explore the ways in which these two relatively nascent fields complement each other, and where they diverge. Our intention was to highlight ways in which these two fields, with their different disciplinary backgrounds, may support further re-thinking of conservation through their shared emphasis on relational approaches and ontological underpinnings. To ground this exploration, and re-think with animals, felids are entangled within this text. Their stories and entanglements with humans outline the scope and implications of what a combined understanding of multispecies studies and compassionate conservation can do for conservation. By combining these two bodies of scholarship to rethink conservation of felids, we identified four key areas for further exploration: (1) A shift in emphasis away from practices of killing to an exploration of the assumptions that make forms of killing permissible and ethically unproblematic, particularly of what are deemed as undesirable others, such as “feral” cats. (2) Re-engagement with individuals, not just species, in conservation settings (3) Unsettling human exceptionalism through an emphasis on the agency of animals and an ethic involving compassion. (4) In shifting away from killability and acknowledging the ways in which humans co-become with other animals, opening space for grappling with how to live well with other animals through multispecies cohabitation.

The emphasis on cohabitation and animal agency in shaping landscapes and opportunities for interactions opens up a rich context for future research to consider questions such as: What deep and entangled histories shape the situated felid–human communities? What kinds of positive entanglements of felid predators and human societies are possible? What is the effect of animal cultures in shaping felid–human cohabitation? How do these relationships unfold in the context of ecotourism and conservation?

## Figures and Tables

**Figure 1:**
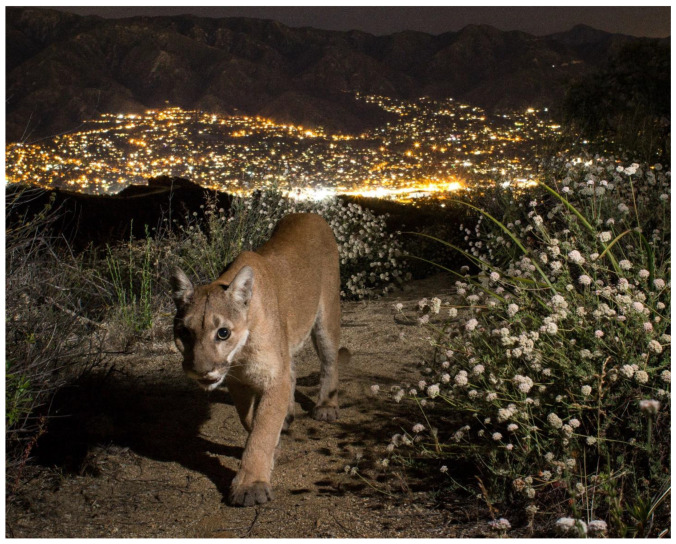
A mountain lion (*Puma concolor*) on the urban fringe of Los Angeles. Image by Joanna Turner.

**Figure 2:**
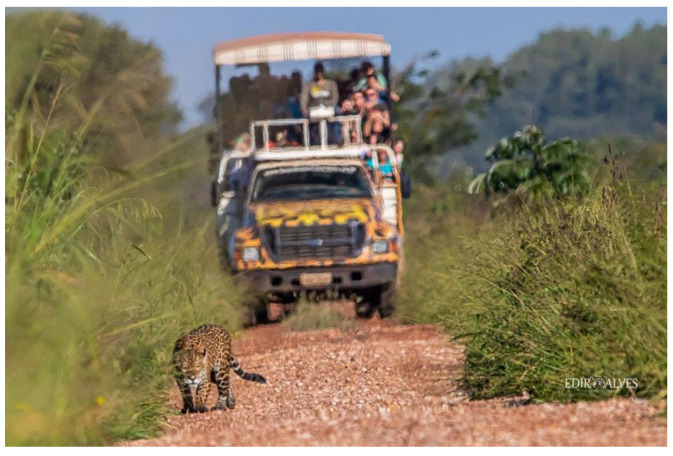
A touristic encounter with a jaguar (*Panthera onca*) at Fazenda San Francisco, Brazil. Image by Edir Alvess.

**Figure 3:**
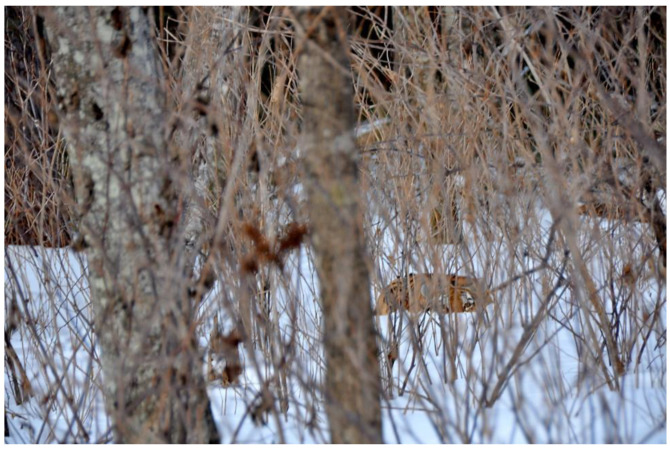
Small scale eco-tourism fosters cohabitation with Siberian tigers (*Panthera tigris altaica*) in the Russian Fareast. Image by Martin Royle (Royle Safaris).
